# COVID-19 Vaccines (Revisited) and Oral-Mucosal Vector System as a Potential Vaccine Platform

**DOI:** 10.3390/vaccines9020171

**Published:** 2021-02-18

**Authors:** Muhammad Umer Ashraf, Yeji Kim, Sunil Kumar, Dongyeob Seo, Maryam Ashraf, Yong-Soo Bae

**Affiliations:** 1 Department of Biological Sciences, Sungkyunkwan University, Jangan-gu, Suwon, Gyeonggi-do 16419, Korea; ashraf@skku.edu (M.U.A.); kyw07043@gmail.com (Y.K.); sunilkumar@skku.edu (S.K.); sdy2089@skku.edu (D.S.); 2 Science Research Center (SRC) for Immune Research on Non-lymphoid Organ (CIRNO), Sungkyunkwan University, Jangan-gu, Suwon, Gyeonggi-do 16419, Korea; 3 Department of Dermatology, Pakistan Air Force (PAF) Hospital, Islamabad 44000, Pakistan; maryamashraf951@yahoo.com

**Keywords:** COVID-19, vaccine candidates, licensed vaccines, humoral/CMI, oral-mucosal vaccine platform, RPS-CTP vector system

## Abstract

There are several emerging strategies for the vaccination of COVID-19 (SARS-CoV-2) however, only a few have yet shown promising effects. Thus, choosing the right pathway and the best prophylactic options in preventing COVID-19 is still challenging at best. Approximately, more than two-hundred vaccines are being tested in different countries, and more than fifty clinical trials are currently undergoing. In this review, we have summarized the immune-based strategies for the development of COVID-19 vaccines and the different vaccine candidate platforms that are in clinical stages of evaluation, and up to the recently licensed mRNA-based COVID-19 vaccines of Pfizer-BioNtech and Moderna’s. Lastly, we have briefly included the potentials of using the ‘RPS-CTP vector system’ for the development of a safe and effective oral mucosal COVID-19 vaccine as another vaccine platform.

## 1. Introduction

The coronavirus disease 2019 (COVID-19) outbreak was first reported in December 2019, in Wuhan, China. Since then, it has spread across the globe making it prevalent in around 216 countries and still counting. This makes COVID-19 the largest pandemic since the 1918 Spanish flu that made a medical nightmare into a reality transcending continents and boundaries [1]. The COVID-19 disease is caused by a positive-stranded respiratory RNA virus known as severe acute respiratory syndrome coronavirus 2, SARS-CoV-2 [2]. Since its outbreak, scientists worldwide are trying to understand the nature and the pathophysiology of this novel coronavirus. Although, our understanding of the SARS-CoV-2 has improved, yet it still needs to be further probed deeper to develop a feasible explanation of how it works and what drives its immunopathology. The COVID-19 disease manifests itself in asymptomatic, mild or severe leading to death [3]. The respiratory tract is the predilection site of the SARS-CoV-2 once it has entered via the mucosal barriers [4,5]. Upon entry, it binds to its receptor angiotensin-converting enzyme type 2 (ACE2) in the respiratory tract (bronchial and epithelial cells) through the receptor-binding domain (RBD) of the spike ‘S’ protein, followed by priming with a specific co-receptor, serine protease known as the transmembrane protease serine 2 (TMPRSS2) [6,7]. It is crucial to understand how this virus elicits immune responses and whereby evades those immune responses to survive and replicate in the body, and this information is vital for the development of a safe and efficient vaccine and/or immunotherapy against the virus. Like MERS-CoV and SARS-CoV, two of the phylogenetically similar viruses, SARS-CoV-2 causes suppression of the innate immune system including the dendritic cells (DCs) and diminishes the antiviral interferons (type I and III IFNs) [8,9,10,11]. Meanwhile, it has also been demonstrated that SARS-CoV-2 elicits an acute hyper-inflammatory response, like cytokine storm, leading to worse prognosis and increased fatality rate (FR) in infected patients [12]. Therefore, both neutralizing antibodies for prevention and cytotoxic T lymphocyte (CTL) responses for inhibition of propagation right after infection are vital to helping fight the COVID-19 infection at an early stage [13,14]. Several vaccine candidates and immune therapies are being developed and evaluated since the COVID-19 outbreak. However, most of the COVID-19 vaccine studies are focused on only one of these two immune responses rather than both. Besides, there are a number of variables that come into play when moving into the clinical phase of the vaccine development especially concerning the safety, dose and dosage and route of administration along with the duration time after vaccination. This review focuses on the various vaccine platforms that are considered potential COVID-19 vaccine candidates in the aspects of both effective prophylaxis and therapeutic potential even after infection. In addition, this review covers an oral mucosal vaccine candidate for the development of a preventative and therapeutic double-targeting SARS-CoV-2 vaccine.

## 2. Discovery/History of Coronavirus

**Figure 1:**
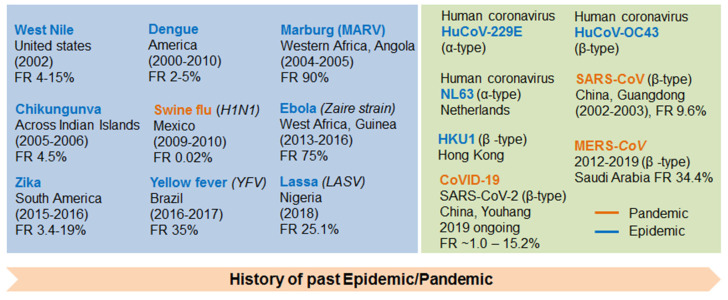
Viral pandemics in the 21st century and their fatality rate (FR) (Upper left)**.** Discovery of human coronaviruses and their FRs (Upper right): Pharmaceutical companies developing COVID-19 vaccine and their clinical stages (Lower).

The current coronavirus, also known as nCoV-19 (Novel Coronavirus-2019) or COVID-19, is not a new type of virus but it belongs to the family of SARS coronavirus [15]. It was first reported in China (November 2002) and named SARS-CoV-1. Since it mainly affects lungs through the respiratory tract, hence named Severe Acute Respiratory Syndrome (SARS). SARS-CoV-1 has affected nearly 29 countries with the total confirmed cases 8096 and 774 deaths with the fatality rate (FR, the proportion of people died with certain disease among the total diagnosed individuals) of 9.6% [16]. This was followed by MERS (Middle East Respiratory Syndrome) outbreak in Saudi Arabia (June 2012), which was also caused by the same family of coronavirus named MERS-CoV (MERS coronavirus). Since then, MERS-CoV has affected 26 countries with confirmed 2519 cases and 866 deaths with an FR of 34.4% [16]. Currently, COVID-19, still spreading, is caused by SARS-CoV-2. It was initially named Novel Coronavirus-2019 (nCoV-2019) and then it was changed to SARS-CoV-2 [17]. On 11 February 2020, the World Health Organization (WHO) renamed the disease as COVID-19 and then announced pandemic in March 2020. It was first reported in Wuhan (China, Hubei province) on December 2019, and so far, it has affected over 213 countries and infected more than 79.8 million people with more than 1.75 million deaths with the FR of 1.0–15.2% worldwide as of 26 December 2020 [18,19]. The history of past epidemics caused by other viruses, for example, West Nile in the United States (2002) with FR 4–15% [20], Dengue in America (2000–2010) with FR 2–5% [21,22], Marburg (MARV) in Western Africa, Angola (2004–2005) with FR 90% [23,24], Chikungunva, Across Indian Islands (2005–2006) with FR 4.5% [25], Ebola (Zaire strain) in West Africa, Guinea (2013–2016) with FR 75% [26], Zika in South America (2015–2016) with FR 3.4–19% [27], Yellow fever (YFV) in Brazil (2016–2017) with FR 35% [28] and Lassa (LASV) in Nigeria (2018) with FR 25.1% [29]. The past pandemics caused by other viruses like Swine flu (H1N1) in Mexico (2009–2010) has been reported to be 12,469 deaths among 60.8 million cases with 274,304 hospitalizations with the FR of 0.02% in the U.S. alone [30,31,32]. The other epidemics caused by a family of coronaviruses includes human coronavirus, HuCoV-229E (α-type) [33], HuCoV-OC43 (β-type) [34], NL63 (α-type) the Netherlands and HKU1 (β-type), Hong Kong [33] are also summarized in Figure 1.

## 3. COVID-19 (SARS-Cov-2) Vaccine Platforms

**Figure 2:**
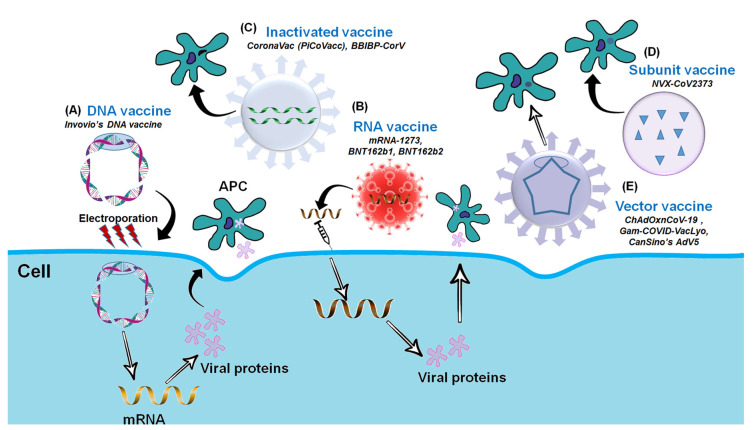
Platforms for the COVID-19 Vaccine Development. (**A**) DNA vaccine: Plasmid DNA expressing S protein. (**B**) RNA vaccine: mRNA-based (RBD of S-protein). (**C**) Inactivated vaccine: Inactivated whole SARS-CoV-2. (**D**) Subunit vaccine: Recombinant S-protein and (**E**) Vector-based vaccine: Replicating or Non-replicating viral vector used for the delivery and expression of S protein.

Following vaccine platforms are being used for COVID-19/SARS-CoV-2 vaccine development (Figure 2).

### 3.1. Inactivated or Killed Vaccines

Inactivated or killed vaccines, as the name suggests, are vaccines in which the virulent agent e.g., infectious virus is killed or inactivated either by chemical or physical means [35,36]. In the case of SARS-CoV-2, the virus usually grown on Vero cells (established cell line from African green monkey kidney epithelial cells) is chemically inactivated [37,38]. Many human vaccines successfully used against Influenza, Hepatitis A and poliomyelitis were killed or inactivated vaccines [39,40,41,42]. Unlike their counterparts, these inactivated or killed vaccines are safer to use, which is a major concern in the case of SARS-CoV-2 vaccines [43,44]. Apart from being safer, these inactivated or killed vaccines express surface antigens which retain their epitope conformations to play an important role in inducing strong preventative humoral responses especially with reference to SARS-CoV-2 [45,46]. Their production is relatively easier but limited in terms of the yield due to reduced SARS-CoV-2 viral productivity in cell cultures as well as the biosafety level 3 (BSL3) production facility requirements. The most common route for the administration of these inactivated or killed vaccines is intramuscular (*i.m.*) and are usually adjuvanted with alum, for instance [38,47]. Since the whole virus is presented to the immune system, immune responses are likely to target not only S but also the matrix (M), envelope (E) and nucleoprotein (N). Although, apart from the S protein antigen, the humoral immune responses against M, E and N proteins may have nothing to do with the preventative immunity against COVID-19. Several inactivated vaccines against SARS-CoV-2 are currently being produced and some have even entered in the clinical trials (Chines candidates in Phase III and one Indian, a Kazakh and a Chinese candidate in Phase I/II clinical trials.). Sinovac’s CoronaVac in China [44] is one prime example of inactivated vaccine which is discussed in detail later. Apart from China, India and Kazakhstan and some other countries are also focusing on this vaccine platform for the production of SARS-CoV-2 vaccines [48]. 

### 3.2. Live-Attenuated Vaccines

Unlike inactivated or killed vaccines, activated or live-attenuated vaccines use the live but weakened or attenuated strain of the virulent agent [49]. This weakened or attenuated agent has the ability to replicate but to a limited extent so that it can elicit or mimic an immune response as would be in case of natural infection but without causing the disease itself [50]. This attenuation is achieved either by continuous passage either in vitro or in vivo of the virulent agent by exposing it to unfavorable conditions or by means of genetic manipulation like mutagenesis (e.g., modifying the virus by deleting viral genes essential for replication, host-tropism, immune evasion or invasion or by codon de-optimization) [51,52,53,54,55,56]. Several successful human vaccines have been made using this live attenuated vaccine platform including the Bacillus Calmette-Guérin (BCG) vaccine for tuberculosis (TB), the Measles vaccine, oral polio vaccine (OPV), and live attenuated seasonal influenza vaccine [54,57,58,59,60,61]. Except for BCG and measles vaccines, an important feature of these live attenuated vaccines is that they can be administered by routes other than parenteral i.e., oral or intranasal routes. This intranasal route of vaccine delivery can protect the upper respiratory tract which in SARS-CoV-2 case is the major portal of entry [62,63,64,65]. Furthermore, since the attenuated vaccine agent is replicative inside the subject, it can induce both humoral and cellular immune responses by presenting foreign proteins of infectious agent expressed in the cytoplasm to adaptive immune cells. However, unlike their inactivated counterparts, this live vaccine platform is at a disadvantage of being less safe due to the live nature of the virus. Besides, it is still not well- established which components or genes of SARS-CoV-2 are critical or essential for the fatal symptoms of COVID-19 after infection. Moreover, the generation of genetically modified and live attenuated vaccine candidates with such a big RNA virus-like SARS-CoV-2 is itself another laborious time-consuming job, to say the least [66,67,68,69]. This seems to be the reason that only three live-attenuated vaccines are in preclinical trials including the one developed by Codagenix Inc. in collaboration with the Serum Institute of India [48]. Recently, a live-attenuated vaccine against SARS-CoV-2 was developed by gradually adapting the SARS-CoV-2 strain (SARS-CoV-2/human/Korea/CNUHV03/2020) from 37 °C to 22 °C in Vero cells and its preclinical outcomes were reported [70]. Interestingly, even a single dose of this cold-adapted SARS-CoV-2 vaccine administered by the intranasal route was able to induce both high titers of neutralizing antibody (>640 along with mucosal IgA) and cellular immune responses in immunized K18-hACE2 mice with no systemic weight loss or lung disease pathologies [70]. These findings instigate the importance of these live-attenuated vaccines since the ACE2 receptor is highly concentrated in the oro-nasal epithelia whereby the viral entry and early replication occur [71].

### 3.3. Recombinant Vaccines

Recombinant vaccines employ recombinant technology to use one to multiple antigens to induce the immune response against the pathogen in question. This feat can be achieved in a number of ways including subunit vaccines or expression vector-based vaccines using delivery vectors like plasmids or viral/bacterial vectors [72,73,74,75,76]. In the case of recombinant viral vector-based vaccines, a viral backbone, which is either replication-deficient or replication-competent, is engineered to express the target-pathogen-derived antigens. This vaccine platform is widely investigated for the development of vaccines like Ebola due to its strong CTL (cytotoxic T lymphocyte) immunogenicity and safety [77,78]. On the other hand, FluBlok vaccine for influenza is an example of a successful recombinant subunit vaccine [79,80,81,82,83]. In the case of SARS-CoV-2, recombinant vaccines can be classified into VLP (virus-like particle) vaccines, recombinant ‘S’ subunit vaccines and recombinant RBD vaccines which can be manufactured in a variety of eukaryotic expression systems including insect cells, mammalian cells, yeast, plants and in case of RBD, expressed even in prokaryotic *E.coli* [48,52,84]. Another advantage of this platform apart from the CTL response is that they can be produced without handling live viruses [85,86,87]. However, there are some limitations in developing the recombinant vaccines. For instance, the spike ‘S’ protein has a relatively low yield since it is hard to express, which begs the “number of doses” question consequently. On the other hand, RBD peptide is easier to express, but despite its potent immunogenicity [88], it is lacking other neutralizing epitopes which are otherwise present on the S protein vaccines, thus more prone to the antigenic drift than their spike counterparts. Many recombinant vaccines are currently being produced and evaluated in pre-clinical stages in several countries based on either of these protein systems (S/RBD) [89,90,91]. Of those, Novavax (described below) has reported non-human primate (NHP) and Phase I data (NCT04368988). One VLP vaccine, produced by Medicago Inc, has also entered clinical trials Similar to inactivated vaccines, these candidates are typically injected and thus are not expected to induce effective mucosal immunity as well as strong T cell responses (NCT04450004) [92]. 

### 3.4. Nucleic Acid-Based Vaccines

Nucleic acid-based vaccines are vaccines that use nucleic acid either DNA or RNA as a source of antigen against certain pathogens. These DNA or RNA vaccines were devised as an alternative to live or subunit vaccines (which are grown in eggs or cells *ex-vivo)* and are thereby stable, cost-effective, cheaper and developed more quickly [93]. Recombinant DNA vaccines have been in development for quite some time however mRNA vaccines have been recently used as a promising vaccine platform [94,95]. COVID-19 DNA vaccines are plasmid DNA vector-based vaccines encoding the SARS-CoV-2 ‘S’ gene. Despite the high yield of production, DNA vaccines fall short when it comes to immunogenicity compared to their live vaccine counterparts. Therefore, they require booster doses and special intracellular delivery systems (electroporation) to achieve the required effect in-vivo. There are several DNA vaccines that are currently in clinical trial stages for SARS-CoV-2 vaccine (Table 1) [48]. On the other hand, RNA vaccines can be divided into two parts: modified mRNA and self-replicating RNA. These two technologies are utilized to deliver the antigen’s genetic information instead of the antigen itself [96]. The difference between the two RNA technologies comes in terms of number of doses i.e., mRNA requiring high doses as compared to the self-replicating RNA. These are usually delivered by lipid-based nanotechnology known as LNPS or lipid nanoparticles [97,98,99,100]. Considering the recently licensed mRNA vaccines for COVID-19, the RNA vaccine platform seems quite promising in other viral diseases such as influenza, Zika virus and rabies. [101,102,103,104,105]. There are several RNA-vaccine candidates in development for SARS-CoV-2 vaccine (Table 1). Pfizer and Moderna have RNA vaccines in the clinical phase III trials which have recently been granted emergency-use authorization (EUA) by the US FDA [106,107] while other candidates by Arcturus, Curevac, the Imperial College and the Chinese Liberation Army are in Phase I/II of clinical trials [48,108,109]. Although there seems to be not much difference between DNA and RNA vaccines when it comes to immunogenicity, however, the RNA (mRNA or RNA) vaccines have one key advantage since DNA vaccine needs an additional transcription step in in-vivo systems. These nucleic acid-based vector vaccines are safe and are easy to produce but require multiple doses along with a special delivery system due to its poor immunogenicity by itself [110]. A key advantage of these mRNA vaccines is that they can be easily synthesized in the lab provided the target viral protein is known, which in SARS-CoV-2 case, is mostly the spike ‘S’ glycoprotein. This facilitates in generating the specific segments of the ‘S’ protein instead of using the whole protein itself. However, these mRNA vaccines require very low storage temperatures due to their thermolabile nature (unstable at high temperatures) [111]. On the other hand, DNA vaccines offer higher stability over the mRNA vaccines yet the non-integrating nature of the latter reduces the risk of insertional mutagenesis [112], which in COVID-19 case, is very crucial in developing the COVID-19 vaccine since the virus has known to mutate quite rapidly as witnessed with the new mutant UK ‘B.1.1.7’ strain [113]. Additionally, modifications can be made in the mRNA vaccines to enhance the vaccine’s stability and half-life (e.g., Arcturus Therapeutics utilizes the STARR™ technology, which is a self-replicating mRNA combined with the LUNAR^®^ nanoparticle non-viral delivery system) [114] and immunogenicity (Pfizer’s T4 fold-on modification [115]). Furthermore, the use of nanotechnology for vaccine delivery is another feat that has ramifying advantages over the usual adjuvant-based delivery mechanisms [116]. Synthetic nanocarriers (polymeric nanoparticles and cationic liposomes) have been used for DNA vaccine delivery while lipid nanoparticle (LNP) platform has been utilized by Moderna for its mRNA vaccine [35]. 

## 4. SARS-CoV-2 (COVID-19) Vaccines in Clinical Trials (Phase I–III)

In this section, recent clinical trials conducted by several different institutions and pharmaceutical companies for the development of COVID-19 vaccines are summarized in Table 1 and briefly discussed.

### 4.1. AstraZeneca’s ChAdOxnCoV-19 (AZD1222) Vaccine

The University of Oxford together with the Serum Institute of India and AstraZeneca have developed the vaccine candidate based on non-replicating ‘ChAdOx1’ vector which was previously termed as ‘ChAdOxnCoV-19’ and is now known as ‘AZD1222’. AZD1222 vaccine expresses a full-length unmodified wild-type (*wt*) version of the ‘S’ (spike) protein [120]. The advantage of ChAdOx1 vector-based vaccine over commonly used human Ad5 (hAd5) vector-based vaccines is that it is non-human primate-derived i.e., originated from Chimpanzee. Due to its simian origin, the vaccine lacks preexisting vector-mediated immunity which is usually observed in the case of hADv5 vector-based vaccines since most of the human population is seropositive to the hAD5 [129]. Therefore, the AZD1222 vaccine candidate induces strong anti-S antibodies (First dose: median 157 ELISA Units-EU; booster dose: 639 EU) and unhindered neutralizing antibody response [121]. Regarding T cell responses, ChAdOxnCoV-19 vaccine induces Th1 as well as strong CD8^+^ T cell responses and lung resident memory T cells (Trm). The route of administration is parenteral i.e., intramuscular (*i.m.)* and it is being evaluated as a single or two-dose regimen in Phase-III clinical trials in several countries. They recently reported preliminary results from a Phase I/II single-blind randomized control trial in 1077 participants aged 18–55 (NCT04324606) [120,121,130]. The vaccine had mild adverse reactions including chills, fatigue, headache, fever, nausea, muscle aches, malaise, and painful injection sites within a week post-vaccination. As a prophylactic measure, paracetamol was included in the trial protocol to reduce these post-vaccination local and systemic reactions [121]. One patient developed neurological symptoms due to which the phase I/II was paused briefly. Later, however, these neurological symptoms were attributed to the MS (multiple sclerosis). Another patient developed symptoms that were consistent with transverse myelitis in the Phase III trial and the trial was paused consequently. Due to these unforeseeable halts, the US trial has not yet resumed but the UK trial was resumed. Since *AZD1222* vaccine requires refrigeration, it could be problematic for use in under-developed countries [131]. Recently, apart from the UK and Pakistan, five other countries have granted EUA to the AZD1222 vaccine including India, Argentina, Dominican Republic, El Salvador, Mexico and Morocco [132].

### 4.2. Sinopharm’s BBIBP-CorV Vaccine

Another candidate for COVID-19 vaccine is being developed by the Sinopharm, Beijing Institute of Biological Products Co. Ltd. Sinopharm has developed two inactivated vaccines against SARS-CoV-2 and is currently being evaluated in clinical trials in China and UAE in different phases (I–III). Both of these inactivated vaccine candidates use the whole SARS-CoV-2 virus with alum as an adjuvant (ChiCTR2000030906) [38]. Since the whole virus is used, so it uses multiple viral antigens to elicit an effective immune response post-immunization by the parenteral i.e., *i.m.* route. Two-to-three repeated doses are required to produce a sufficient immune response by the *i.m.* route and strong neutralizing dose-dependent antibody titers (GMT: 2 μg, 87.7; 4 μg, 158.9; and 8 μg, 186.1 in 18–59 age group), and (GMT: 2 μg, 80.7; 4 μg, 131.5; and 8 μg, 170.87 in 60 age group) were observed at 42nd day of vaccination [133]. It is termed as Sinopharm’s BBIBP-CorV. Published data from phase I and II trials show that this version of the inactivated vaccine was able to provide protection and humoral immune response by producing neutralizing antibodies. (ChiCTR2000034780, ChiCTR2000032459) [38,44,134]. The local and systemic adverse reactions to the vaccine were comparable to the ‘only alum controls’ [133]. The vaccine has been granted EUA (emergency use authorization) for health-care providers in the UAE [131] and has been administered to hundreds of thousands of people under the EUA condition in China [134].

### 4.3. CanSino’s AdV5-Based Vaccine

Another non-replicating COVID-19 vaccine candidate under development is CanSino’s ADV5-based vaccine. This AdV5 vaccine also expresses the full-length-unmodified spike ‘S’ protein from the Wuhan-Hu-1 virus strain [118]. However, unlike its ChAdOxnCoV-19 counterpart, the durability and the quality of the neutralizing antibody response is affected owing to the pre-existing anti-vector response. Higher anti-RBD antibody geometric mean titers (GMT) were observed on 28th day of vaccination and this titer difference was subject to dose (low, 615.8; medium, 806.0; and high, 1445.8) and day of immunization [118,119]. AdV5, like ChAdOxnCoV-19, also induces both Th1 and strong CTL immune responses however; the pre-existing vector-mediated immunity negatively affects the CTL response. It also induces lung Trm but only by respiratory mucosal (RM) route. The route of delivery is parenteral i.e., *i.m*. Currently, it is being used as a licensed single-dose vaccine in the Chinese military [117]. CanSino reported its Phase-I clinical trial (NCT04313127) and is under evaluation in other Phase-III clinical trials (NCT04341389, NCT04526990, NCT04540419 etc.) [118,119]. Common mild vaccination adverse reactions like painful injection sites, redness, headache, fever, malaise, muscle ache and fatigue were observed within a week of vaccination [119]. No information as to what the storage conditions of the vaccine will be has been published yet but since it is adenovirus vector-based, it is safe to assume that it might need either refrigeration or –20 °C storage. 

### 4.4. Gamaleya’s Sputnik V (Gam-COVID-VacLyo) Vaccine

Russia’s Gameleya National Research Institute for Epidemiology and Microbiology has developed a recombinant non-replicating viral-vectored COVID-19 vaccine. The recombinant vaccine uses Adenovirus as a viral vector and is termed as Sputnik V (formerly known as Gam-COVID-VacLyo). It consists of two recombinant adenoviral serotypes 26 and 5 namely rAD26 and rAD5 respectively. Both of these recombinant viruses carry the spike ‘S’ glycoprotein of the SARS-CoV-2 i.e., rAd26-S and rAd5-S. Sputnik V is currently being evaluated in Phase I and II of clinical trials using either frozen (Gam-COVID-Vac) or lyophilized (Gam-COVID-Vac-Lyo) vaccine formulations (NCT04436471, NCT04437875) [122]. The vaccine is administered by the parenteral route i.e., *i.m.* in single as well as combined with booster dose regimens. Adverse reactions were mild to the vaccine including pain, redness and fever, etc. However, the safety profile, as well as the efficacy of the vaccine, has not been verified in the Phase III trial which raises concerns and which is crucial to take the vaccine into the next step of the evaluation [122]. The published data from clinical trials (Phase I and II) showed that this version of the recombinant-adenovirus vectored, non-replicating vaccine containing the ‘S’ protein was able to produce an immune response which was almost comparable to the one observed in the convalescent plasma of the recovered COVID-19 patients i.e., similar RBD ELISA titers, neutralizing antibodies and CTL responses were produced (NCT04436471, NCT04437875) [122]. The observed anti-RBD IgG titers were increased from GMT values; 3442 and 3442 to 14,703 and 11,143 after booster immunization in both Gam-COVID-Vac and Gam-COVID-Vac-Lyo respectively [122]. The vaccine was approved for use in small population groups by the Institute of Biology at the Academy of Military Medical Sciences before the Phase III trial initiation which has raised many eyebrows within the scientific community [135]. Safety concerns have been raised since the vaccine has not been tested and evaluated in a Phase III clinical trial which is necessary for the vaccine to move forward to the final stages of evaluation.

### 4.5. Novavax’ NVX-CoV2373

Apart from exploiting the unmodified spike ‘S’ protein for a potential COVID-19 vaccine, there is another version of the vaccine that makes use of the recombinant technology with nanoparticle technology [136]. Novavax is developing a recombinant vaccine “NVX-CoV2373”, which employs the full-length spike ‘S’ protein with some modifications (deleted polybasic cleavage site and two proline mutations) leading to rosette-shaped spike with hydrophobic tails. The spike protein is perfused with the saponin-based Matrix-M1 adjuvant (Adjuvant Matrix-M™ is comprised of 40 nm nanoparticles composed of Quillaja saponins, cholesterol and phospholipid). NVX-CoV2373 has undergone Phase-I trial (NCT04368988) which was recently published by Novavax. It is used as a two-dose regimen for vaccination and is currently in the Phase-II trials (NCT04533399) [137]. Results have shown that this adjuvant-based recombinant ‘S’ vaccine can induce neutralizing antibodies and provide protection against SARS-CoV-2 challenge in immunized macaques [137]. Immunized mice and baboons were able to generate T cell immunity (strong CD4^+^ and CD8^+^ CTL responses) and B cells in the antigen-specific germinal center (GC) of the spleen [138]. No serious adverse effects were observed in the first-in-human trials with robust IgG anti-spike geometric mean ELISA units (GMEUs, 63,160) and neutralizing antibodies (GMT, 3906) both of which were four times higher than those observed in the convalescent serum of the COVID-19 clinical patients (GMEUs, 8344 and GMT, 983 respectively). The high titer of neutralizing antibody, although observed in non-human primates, is hoped to be translated in the human trial subjects as well and it seems to be higher than any other vaccine candidates. However, the data are not directly comparable because the vaccines act through different mechanisms, and preclinical tests were conducted under different dosing regimens [136]. Recently, the vaccine has entered into the phase III of clinical trials in the UK (EudraCT 2020-004123-16, NCT04583995), Mexico, Puerto Rico, the US (NCT04611802), South Africa (NCT04533399) and Australia (NCT04368988).

### 4.6. Sinovac’s CoronaVac (PiCoVacc)

Another COVID-19 vaccine under development is the Sinovac’s inactivated SARS-CoV-2 vaccine. They termed the inactivated vaccine as the CoronaVac (PiCoVacc, as per publication). It is a chemically inactivated, whole SARS-CoV-2 preparation [37]. PiCoVacc is being evaluated in Phase I/II/III trials in Brazil and China (NCT04456595) in both adults and geriatric patients. It is required as a two-dose regimen (at 0 and 28 days). The route of inoculation is parenteral i.e., *i.m.* [47,48]. No serious local and systemic reactions to the vaccine were observed [47]. Sinovac was granted EUA by the Chinese government in July 2020 before Phase III initiation which resulted in almost 90% of the company employees being immunized with the vaccine [139]. It was observed that the neutralizing antibody titers were comparatively higher in younger patients to older ones and the second dose kinetics yielded different responses i.e., stronger immune responses with the second dose on 28th day instead of the 14th day (NCT04352608) [47,131]. Anti-RBD antibodies, as well as mean neutralizing antibody titers (GMT, 23.8–65.4), were observed two weeks after the second dose of vaccination. Although these GMT values of the neutralizing antibodies (GMT-maximum, 65.4) induced in response to the vaccination were lower than those observed in the convalescent sera of the recovered COVID-19 patients (GMT-maximum, 163.7), however, it is still regarded as an attractive vaccine candidate for underdeveloped countries owing to its storage conditions (2–8 °C) [131]. Apart from humoral immunity, no data have been provided about the cellular immune responses to the vaccine. 

### 4.7. Johnson & Johnson (J & J)’s Ad26.COV2.S (JNJ-78436735)

Another vector-based recombinant vaccine is being evaluated in the Phase III clinical trial by the Janssen Pharmaceutical Companies of Johnson & Johnson. The vaccine, known as Ad26.COV2.S (JNJ-78436735), is based on a replication-defective human adenovirus serotype 26 that expresses the full-length ‘S’ glycoprotein of the SARS-CoV-2. The Janssen platform used—AdVac vaccine platform- was initially developed for Ebola and later for Zika, RSV, and HIV vaccines [140]. Results have shown that a single non-adjuvanted immunization with J & J’s Ad26.COV2.S vaccine can provide protection against SARS-CoV-2 challenge in rhesus macaques aged 6–12 years by inducing strong neutralizing antibody titers [123]. It can be stored at 2–8 °C for at least three months and has been estimated to remain stable for two years at −20 °C. The vaccine is the Phase I/II clinical trials in the States and Belgium. The Phase III trial was briefly paused due to some serious adverse effects in a single patient post-vaccination but after careful investigation by the independent Data Safety and Monitoring Board (DSMB), the company has been granted permission to resume the Phase III Ensemble trial [140]. According to an interim report on the vaccine’s efficacy and safety (published in NEJM, 2021), the first dose of the vaccine sustained neutralizing antibody titers (GMT, 288–488) till day 71 and the second dose further enhanced the titer values (GMT, 827–1266). Spike-binding antibody responses were similar to neutralizing-antibody responses. No severe adverse effects were observed in the study [141].

## 5. Licensed SARS-CoV-2 (COVID-19) Vaccines

### 5.1. Pfizer-BioNTech’s BNT162b2 Vaccine

mRNA-based COVID-19 vaccine was developed in a collaborative effort by Pfizer and German company BioNTech and approved most recently for emergency-use. This mRNA-vaccine has two versions: BNT162b1 and BNT162b2 both developed by Pfizer-BioNTech. BNT162b1 is a lipid-nanoparticle (LNP)-delivered mRNA vaccine that expresses the RBD (trimeric form) of ‘S’ protein held together by a T4-foldon (the natural trimerization domain of T4 fibritin) (NCT04368728) [115]. On the other hand, BNT162b2 expresses a full-length spike subunit protein with two proline mutations. Pfizer recently published a comparative analysis of these two vaccine candidates and while these two were quite comparable to antibody titers and the CD4^+^/CD8^+^ T cell responses, the safety profile of the later i.e., BNT162b2 was favorable. Due to the safety concerns, BNT162b2 was moved to the next stage of the clinical trials (NCT04368728) in both adults and the elderly subjects [109,115,142]. Following Phase-III trial (the results have not been published yet), Pfizer and BioNTech have submitted their COVID-19 vaccine candidate (code-named BNT162b2, commonly known as the Pfizer–BioNTech COVID-19 vaccine, and sold under the brand name Comirnaty) to the US Food and Drug Administration (FDA) for emergency-use authorization in November 2020 [107]. The FDA panel issued an emergency use authorization (EUA) for the Pfizer-BioNTech COVID-19 (BNT162b2) vaccine on 11 December 2020, and has approved the vaccine for emergency-use [143]. However, the UK had already approved the vaccine for use prior to the FDA’s authorization [144]. Pfizer and BioNTech have reported 95% efficacy in the Phase III trial of BNT162b2 [145]. According to a report published in the New England Journal of Medicine (NEJM), the efficacy of the said vaccine was 52% after the first dose but it was significantly increased to 95% following the second dose. Therefore, a two-dose vaccination regimen is recommended against COVID-19 in people aged 16 years or older [146,147]. Despite its overwhelming efficacy (>90%) across all demographics, the vaccine is advised to be used with caution owing to certain side-effects in specific people. According to The Medicines and Healthcare Products Regulatory Agency (MHRA), it has been advised to administer the vaccine with caution to individuals who have any history of an allergic reaction to a vaccine, drug or food and especially people who need an adrenaline auto-injector in emergency cases if need be. The MHRA also insists on the availability of resuscitation facilities for all vaccinations. This potential anaphylactic response was observed in two UK patients who were vaccinated with BNT162b2 vaccine [148].

### 5.2. Moderna’s mRNA-1273 Vaccine

Another licensed vaccine for COVID-19 is mRNA-based “Moderna’s mRNA-1273 vaccine”. The mRNA-1273 encodes the full-length spike ‘S’ protein with two stabilizing mutations and is encapsulated in lipid nanoparticles (LNPs) for delivery and the adjuvant potential (NCT04470427, NCT04283461, NCT04405076) [97,108]. Due to the lack of pre-existing anti-vector immunity, the neutralizing antibody response is unimpeded. It is administered via the parenteral (*i.m.)* route in a two-dose regimen given at 4 weeks apart. In the Phase I trial (NCT04283461), strong dose-dependent anti-S-2P antibody titers were observed after first immunization at 25 μg and 100 μg doses among the age groups of 56–70-year-old and 71 or above, the observed GMT values were 323,945; 1,183,066 and 1,128,391; 3,638,522 respectively. Following the booster dose, virus-neutralizing antibody titers were observed across young and old patients. These humoral responses were almost similar to the ones observed in the convalescent plasma of patients who had recovered from COVID-19 [149]. Although good CD4^+^ T cell responses were measured post-vaccination, however, the CTL responses were low as expected for the spike protein. Only high doses caused adverse effects which were reduced in low dose regimen. The painful injection site, fever, chills and myalgia were observed within a few days of vaccination and no safety concerns were observed with this vaccine [150]. Storage condition for the vaccine is –20 °C which might be a problem for vaccine deployment [131]. This mRNA-1273 vaccine is being evaluated in adults and geriatric patients in Phase-III clinical trials (NCT04470427) [99,108,149,151]. Phase III trial was conducted and it was stated that the mRNA vaccine showed 95% efficacy across all demographics and Moderna has sought EUA from the US FDA in November 2020 [152]. The US FDA has granted EUA to Moderna on 18 December 2020, which will allow the Moderna’s mRNA-1273 vaccine to be distributed in the US for use in individuals of 18 years of age and older [106].

## 6. Mucosal Vaccines-Platform for COVID-19 Vaccine Development

According to the guideline of The World Health Organization (WHO), most of the COVID-19 vaccines are designed to be delivered by the parenteral intramuscular route to produce high titers of systemic neutralizing antibody to cope with the systemic viral infection [48]. However, this strategy leaves some questions about the durability and efficacy of the mucosal immune response after vaccination which is essential for blocking the viral entry through oro-respiratory tracts. Despite the dependence on the intramuscular approach, mucosal-based immunizations have been well established for preventative immunity against several respiratory infectious diseases from antiquated through modern times [153]. The late Norwegian immunologist, Per Brandtzaeg was a vocal advocate of mucosal routes of immunization including the oral and intranasal routes partly because he believed that the adenoids and tonsils of the upper airway tract (URT) were responsible to provide both mucosal and systemic immunoglobulins i.e., IgA and IgG antibodies [154]. This potential protective cover provided by the oral-nasal URT might be able to explain why the SARS-CoV-2 leads more casualties in the elderly compared to the younger groups. Since the COVID-19 epitomizes the mucosal disease process, SARS-CoV-2 not only gains access to the host by mucosal routes but these mucosal sites seem to be the predilection sites for the virus where it resides predominately. These oronasal and conjunctival mucosa provide the transmission routes for the SARS-CoV-2 through aerosol droplets, close contact or fomites. The virus enters via the mucosal barriers and thereby invades the underlying mucosal and epithelial layers of the respiratory tract (lungs). It has recently been shown that a high expression of SARS-CoV-2 receptor, ACE2 is observed in the mucosal linings of the enterocytes of the digestive system i.e., in the ileum and colon [155] Interestingly, ACE2 is found to be highly concentrated in the oronasal epithelium and the lowest in the alveoli [156]. This suggests that viral replication is profound in the mucosal sites (oral/nasal) compared to that in the alveoli [71]. These findings support the idea of an oral/nasal mucosal vaccine against SARS-CoV-2; since in nature oral vaccines are well established to induce and activate the common mucosal immune system and therefore have been successfully used for enteric and respiratory infectious diseases before [157]. The live oral enteric-coated adenovirus (AV) vaccines (type 4 and 7) approved for use in the US military have shown to be tremendously effective [158]. The oral tablet versions of the COVID-19 vaccine as discussed below would be used to reach regions where proper health-care or professionals are not available especially in the under-developed countries. The tablet form of the vaccine would enable the common person who has no reach to a proper vaccination site without having to need any health-care professional or the fear of the common side-effects of the injectables such as malaise, pain, and inflammation.

### 6.1. Vaxart’s Oral Mucosal COVID-19 Vaccine

Vaxart has recently developed an oral recombinant COVID-19 vaccine tablet that has moved to the Phase-I trial (NCT04563702). The enteric-coated tablet vaccine contains an adenoviral-vector that encodes the genes for spike ‘S’ and the nucleocapsid ‘N’ proteins of the SARS-CoV-2. The enteric coating prevents the tablet’s active ingredient from the stomach’s acidic environment. These coated tablets dissolve in the digestive tract providing protective mucosal immunity against the viral infection. Vaxart reported that hamsters that received a two-dose regimen (at 0 and 4 weeks) of the oral vaccine showed neither systemic weight loss nor lung disease symptom, which is usually observed in non-protected hamsters. All animals vaccinated with the Vaxart vaccine showed a significant increase of neutralizing antibodies against SARS-CoV-2 compared to the non-vaccinated group in the serum two weeks after the first vaccination [159]. According to the pre-clinical report published by Vaxart, the full-length wild-type (*wt*) spike ‘S’ protein antigen, when administered mucosally, induces higher neutralizing antibody titers compared to that of S1 domain or stabilized ‘S’ antigen both in the lungs and in the periphery. Both low and high doses of the vaccine tablet were able to induce the antigen-specific CD4^+^ and CD8^+^ T cells. Additionally, the recombinant adenoviral vector vaccine incorporating the full-length ‘S’ and ‘N’ antigens is underway to move to the clinical phase of evaluation [160]. This opens new doors to the development of oral mucosal vaccines for SARS-CoV-2. Recently, two vaccines have gained quite the attention in terms of COVID-19 vaccine platforms: the BCG vaccine against TB and the OPV (oral polio vaccine) against poliomyelitis. It has been suggested that these pre-existing oral vaccines can ameliorate the COVID-19 effects in patients via broader protection against unrelated pathogens likely by inducing interferon (IFN) and other innate immune mechanisms that are yet to be identified [161,162].

### 6.2. IosBio’s (Sabilitech’s) OraPro-COVID-19™ Vaccine

Another oral vaccine candidate under development by a UK-based company isoBio (previously known as Stabilitech) is OraPro-COVID-19™ [163]. As of June 2020, the company has started its collaboration with BioCell Corporation (New Zealand) to manufacture its oral coronavirus vaccine, OraPro-COVID-19 [164]. It uses non-replicating viral-vector that expresses the ‘S’ protein and is used as a thermally stable capsulated form [164]. A replication-defective adenovirus-5 (Adv5) vector encoding the ‘S’ glycoprotein of the SARS-CoV-2 is enteric-coated (encapsulated) and delivered orally directly to the intestinal lymphoid tissues generating both humoral (antibody-mediated) and cellular (CD4^+^ and CD8^+^ T cell-mediated) immune responses with impeded anti-vector immune response [163]. The self-administered capsulated vaccine, if passes the clinical phases of evaluation, would be a great achievement to immunize millions of people around the globe without the need of any assistance from a health-care professional. In addition, since it is provided as a thermally-stable capsule, there is no need of refrigeration which could be a major problem with many other vaccine candidates that need a lower temperature for storage and deployment, especially in low-income countries. The company has not yet published any safety and efficacy profile of the vaccine and has previously used its ORAPRO™ (enteric-coated recombinant adenovirus vector-rAdv) platform for Zika virus vaccination [164]. 

### 6.3. Broad-Spectrum of Pre-Existing Mucosal Vaccines

Mucosal immunity plays a critical role in the inhibition of viral entry through oral route or respiratory tract. Recently, two vaccines have gained quite the attention in terms of COVID-19 vaccine platforms: the BCG vaccine against TB and the oral polio vaccine (OPV) against poliomyelitis. It has been suggested that these pre-existing mucosal and oral vaccines can ameliorate the COVID-19 effects in patients via broader protection against unrelated pathogens likely by inducing interferon (IFN) and other innate immunity that are yet to be identified [161]. Unlike IPV, OPV has been used as the most effective successful preventative vaccine against poliomyelitis via induction of poliovirus-specific mucosal immunity [5]. In addition, more neutralizing antibodies were detected in the nasopharyngeal of OPV-treated patients than in IPV-treated ones [6]. Most mucosal immunity tends to decrease over time, but OPV can prevent reinfection by maintaining a mucosal immune response in the intestine [165].

### 6.4. RPS-Vector System as A Potential Platform for COVID-19 Oral Mucosal Vaccine 

**Figure 3:**
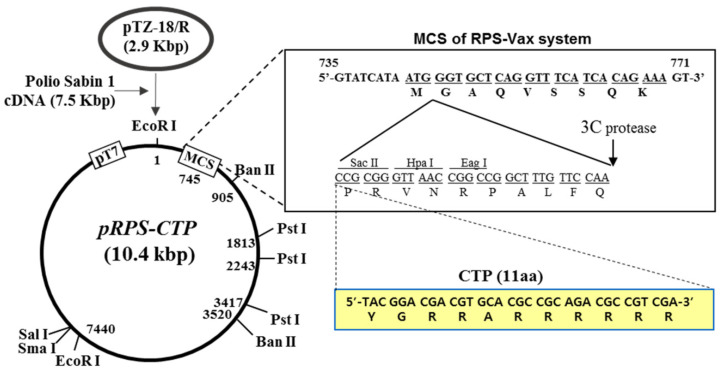
RPS-vector system as a potential platform for COVID-19 oral mucosal vaccine (Seung-Soo et al., 2015).

In this section, Sabin-1 poliovirus cDNA-based RPS (recombinant poliovirus Sabin 1) -vector system (Figure 3) is discussed as a potential platform for the development of an effective oral mucosal COVID-19 vaccine. The RPS-vector system is a vector. Sabin-1 is one of the three attenuated poliovirus serotypes (OPV) and is considered to be developed as a safe and effective mucosal vaccine vector. The RPS-vector system has two versions: the RPS-Vax and the RPS-CTP. The RPS-Vax is a recombinant Sabin-1 poliovirus vector system that contains the multiple cloning site (MCS) and the 3C-protease cutting site for cloning a vaccine gene and release the vaccine protein from the viral particle during their replication, respectively [166]. RPS-Vax vector system was designed and constructed for the development of the mucosal vaccine by exploiting the special characteristics of the OVP vaccine.

On the other hand, the RPS-CTP vector system is a modified version of the RPS-Vax vector harboring CTL-inducible cytoplasmic transduction peptide (CTP) right above the MCS. RPS-CTP vector-based recombinant poliovirus induces antigen-specific CTL responses by exploiting the CTP technology which delivers the CTP-fused vaccine protein, expressed during the rec-poliovirus replication in the payer’s patch, into the cytoplasm of the adjacent cells followed by presentation of CTP-fused antigen through MHC class I [166]. When HIV-1 p24 was incorporated into RPS-CTP vector system, the recombinant poliovirus vRPS-CTP/p24 was effective to induce high titers of p24-specific neutralizing IgA and p24-specific strong CTL responses as well in Tg-PVR mice [166]. The vaccine efficacy of the vRPS-CTP/p24 was examined in the challenge experiment with recombinant vaccinia virus expressing HIV-1 p24 (recVV-p24). Body-weight and survival rate were least affected, and the titer of the recVV-p24 in the lung significantly decreased in mice vaccinated orally with the vRPS-CTP/p24 [166]. Since the RPS-CTP platform-based mucosal vaccine was designed to be administered through the oral route instead of the commonly used parenteral or intramuscular routes, RPS-CTP-derived mucosal vaccine has several advantages of preventing potential side-effects of injection ones and vaccine-loss during the administration especially in geriatric patients which are mostly the victims of the SARS-CoV-2 [167,168,169]. In addition, the rec-poliovirus vRPS-CTP/p24 was genetically stable over 12 passages [166]. It is also well established that the OPV induces long-term T cell and B cell memory [170]. Collectively, these results strongly suggest that the established RPS-CTP vector system can be used as a potential platform technology for the development of preventative and therapeutic mucosal vaccines against COVID-19 and other pandemic viral diseases. 

## 7. Conclusions

The COVID-19 pandemic has left the scientific community with many open questions since it has taken almost a year to license a potential COVID-19 vaccine. This pandemic provided the scientists around the globe with the opportunity to divulge into many facets of the immunology including deep machine learning, genomics, virus surveillance, etc. and paved the way to provide a suitable protective vaccine as a united front. Although, we have learned a lot about the new coronavirus (SARS-CoV-2) and its immunity induced by vaccines through these new technological advancements, still much remains to be learned about its immunopathology, for instance, as to how this virus is able to evade the immune response and mutate as a consequence. A new mutant COVID-19 strain named as B.1.1.7 has recently emerged in the UK (8 December 2020), which has been speculated to cause a surge in both the number of COVID-19 cases as well as in the severity of the disease itself. This mutant strain is better adapted to spreading the previous strain and has known to acquire 17 mutations all at once, a feat never seen before. This causes concerns regarding the efficacy of the recently-licensed vaccines (Pfizer-BioNTech, Moderna) in December 2020 and their protective cover against the new emerging COVID-19 mutant strains. There is hope that the present vaccines and the many other vaccine candidates in the process of the ongoing preclinical and clinical trials around the globe might provide a singular protective vaccine against the COVID-19 and its emerging variants as an extension. However, much is still in the unknown as to what factors account into the viral-mutagenicity and how this impacts different demographics in terms of age, sex, race and ethnicity, not to mention the special cases including the immunocompromised and pregnant patients. Considering these difficult aspects, there is a view among some in the scientific community that a single vaccine might not be the solution to this situation in particular. Therefore, in light of these findings, we have summarized current platforms for the development of COVID-19 vaccine in this review. We have highlighted the possibilities of using different kinds of vaccines targeting crucial SARS-CoV-2 genes, which in turn can be utilized in controlling COVID-19 spread, that simultaneously boost the patient’s immune system to fight subsidiary infections. Moreover, we have shed light on the on-going clinical trials of COVID-19 vaccines and the conducting institutes and pharmaceutical companies involved. This review provides a better understanding of current platforms for COVID-19 vaccine development and the potential use of the RPS-CTP vector system for the development of oral mucosal COVID-19 vaccine as a new vaccine platform for future challenges.

## Figures and Tables

**Table 1:** Current COVID-19 vaccines in clinical trials.

Company/ Organization	Brand Name	Vaccine Type/Platform	Phase	Country	Reference/Trial Identifier
**Inactivated/Killed Vaccines**					
Bharat Biotech, Indian Council of Medical Research, National Institute of Virology	Covaxin	Inactivated SARS-CoV-2 (multiple viral antigens)	I/II	India	NCT04471519
Chinese Academy of Medical Sciences	COVID-19 vaccine	Inactivated SARS-CoV-2 (multiple viral antigens)	I/II	China	NCT04470609, NCT04470609, NCT04412538
Sinovac Biotech	CoronaVac (PiCoVacc)	Inactivated SARS-CoV-2 (multiple viral antigens)	I/II/III	China, Brazil	[37] NCT04456595, NCT04383574, NCT04352608
Sinopharm, Beijing Institute of Biological Products Co. Ltd	BBIBP-CorV	Inactivated SARS-CoV-2 (multiple viral antigens)	I/II/III	China	[38] ChiCTR2000030906
**Live attenuated Vaccines**					
-	-	-	-	-	-
**Recombinant Vaccines**					
CanSino Biologics Inc., Beijing Institute of Biotechnology	Ad5-nCoV	Non-replicating adenoviral (Ad5) vector	I/II/III	China, Canada, Russia	[117,118,119] NCT04313127 NCT04313127 NCT04341389
AstraZeneca, University of Oxford, Serum Institute of India	ChAdOxnCoV-19 (AZD1222)	Non-replicating viral vector (ChAdOx1)- expressing S protein	I/II	UK, South Africa USA, Brazil	[120,121]
Gameleya Research Institute	Sputnik V (Gam-COVID-VacLyo)	Recombinant non-replicating viral (Ad5- or Ad26)-vectored	I/II	Russia	[122] NCT04436471, NCT04437875
Johnson & Johnson	Ad26.COV2-S (JNJ-78436735)	Ad26-vectored, non-replicating, nanoparticle	I/II	USA, Belgium	[123] NCT04436276
Merck, IAVI	COVID-19 vaccine	VSV-vectored, replicating	I/II	USA, Austria, Belgium	NCT04498247, NCT04498247
Novavax	NVX-CoV2373	Recombinant S-protein	I/II	Australia	NCT04368988
**DNA-based Vaccines**					
AnGes Inc., Osaka University, Takara Bio	AG0302-COVID19	Plasmid DNA (expressing S protein)	I/II	Japan	NCT04527081, NCT04527081
Entos Pharmaceuticals	Covigenix VAX-001	Plasmid DNA (expressing S protein)	I/II	Canada, USA	NCT04591184
Genexine Consortium	GX-19	Plasmid DNA (expressing S protein)	I/II	South Korea	NCT04445389
Inovio Pharmaceuticals, International Vaccine Institute	NO-4800a	Plasmid DNA (expressing S protein)	I/II/III	USA	[124] NCT04336410, NCT04447781
Zydus Cadila	ZyCov-D	Plasmid DNA (expressing S protein)	I/II	India	CTRI/2020/07/026352, CTRI/2020/07/026352
**RNA-based Vaccines**					
Academy of Military Medical Sciences, Walvax Biotechnology, Suzhou Abogen Biosciences	ARCoV	mRNA (expressing S protein)	I	China	[125] ChiCTR2000034112
Arcturus Therapeutics, Duke-National University of Singapore	Lunar-COV19	Self-replicating mRNA (expressing S protein)	I/II	Singapore	NCT04480957 NCT04480957 NCT04480957
CureVac	CVnCoV	Lipid nanoparticle- mRNA	I	Germany, Belgium	NCT04449276
Imperial College London, Morningside Ventures	LNP-nCoVsa- RNA	Lipid nanoparticle- saRNA (expressing S protein)	I/II	UK	[126,127] ISRCTN17072692
Moderna, NIAID (VRC)	mRNA-1273	mRNA-based (Lipid nanoparticle– mRNA)	III	USA	[97,108] NCT04283461, NCT04470427, NCT04405076
Pfizer, BioNTech, Fosun Pharma	BNT162b1, BNT162b2	mRNA-based (RBD of S-protein)	I/II/III	Germany, USA, China	NCT04368728
**Subunit Vaccines**					
Anhui Zhifei Longcom Biologic Pharmacy, Chinese Academy of Medical Sciences	COVID-19 vaccine	Protein subunit (dimeric RBD)	I/II/III	China	NCT04445194 NCT04466085 NCT04646590
Clover Pharmaceuticals, GlaxoSmithKline, Dynavax	SCB-2019	Protein subunit (trimeric S protein)	I	Australia	[128] NCT04405908
Kentucky Bioprocessing Inc.	KBPCOVID-19	Protein subunit (RBD-protein)	I/II	USA	NCT04473690
Medicago, Laval University	COVID-19 vaccine	Virus-like particle (VLP)	I	Canada	[92] NCT04450004
Medigen Vaccine Biologics, Dynavax	MVC-COV1901	Protein subunit (S-protein)	I	Taiwan	NCT04487210
University of Queensland	COVID-19 vaccine	Protein subunit (molecular Clamp-stabilized S-protein)	I	Australia	NCT04495933
Vaxine Pty Ltd, Medytox, Central Adelaide Local Health Network	COVAX19	Protein subunit (S-protein with Advax-SM adjuvant)	I	Australia	NCT04453852

## Data Availability

Not applicable.

## Abbreviations

ACE2: angiotensin-converting enzyme 2, AV; adenovirus, BCG; Bacillus Calmette-Guérin, CMI; cell-mediated immunity, CTP; cytoplasmic transduction peptide, CTL; cytotoxic T lymphocyte, COVID-19; coronavirus disease-2019, DSMB; Data Safety and Monitoring Board, E; envelope, EUA; emergency-use authorization, EU; ELISA units, FR; fatality rate, Food and Drug Administration (FDA), GMEUs; geometric mean ELISA units, GMT; geometric mean titer, i.m.; intramuscular, IFN; interferon, IPV inactivated polio vaccine, LNPs; lipid nanoparticles, LASV; Lassa mammarenavirus, M; matrix, MSC; multiple cloning site, MERS; Middle East respiratory syndrome, nCoV-19 (Novel Coronavirus-2019), New England Journal of Medicine (NEJM), N; nucleoprotein, OPV; oral polio vaccine, RBD; receptor binding domain, RPS; recombinant poliovirus Sabin-1, recVV; recombinant vaccinia virus, SARS-CoV-2; severe acute respiratory syndrome-coronavirus type 2, S; spike protein, TMPRSS2; transmembrane protease serine 2, Trm; resident memory T cell, URT; upper airway tract, VLP; virus-like particle, WHO; World Health Organization, YFV; Yellow fever, J&J; Johnson & Johnson.

## References

[1] Trilla A., Trilla G., Daer C. (2008). The 1918 “Spanish Flu” in Spain. Clin. Infect. Dis..

[2] Zhu N., Zhang D., Wang W., Li X., Yang B., Song J., Zhao X., Huang B., Shi W., Lu R. (2020). A Novel Coronavirus from Patients with Pneumonia in China, 2019. N. Engl. J. Med..

[3] Chau N.V.V., Thanh Lam V., Thanh Dung N., Yen L.M., Minh N.N.Q., Hung L.M., Ngoc N.M., Dung N.T., Man D.N.H., Nguyet L.A. (2020). The natural history and transmission potential of asymptomatic SARS-CoV-2 infection. Clin. Infect. Dis..

[4] Sungnak W., Network H.L.B., Huang N., Bécavin C., Berg M., Queen R., Litvinukova M., Talavera-López C., Maatz H., Reichart D. (2020). SARS-CoV-2 entry factors are highly expressed in nasal epithelial cells together with innate immune genes. Nat. Med..

[5] Zou X., Chen K., Zou J., Han P., Hao J., Han Z. (2020). Single-cell RNA-seq data analysis on the receptor ACE2 expression reveals the potential risk of different human organs vulnerable to 2019-nCoV infection. Front. Med..

[6] Hoffmann M., Kleine-Weber H., Schroeder S., Krüger N., Herrler T., Erichsen S., Schiergens T.S., Herrler G., Wu N.-H., Nitsche A. (2020). SARS-CoV-2 Cell Entry Depends on ACE2 and TMPRSS2 and Is Blocked by a Clinically Proven Protease Inhibitor. Cell.

[7] Sallenave J.-M., Guillot L. (2020). Innate Immune Signaling and Proteolytic Pathways in the Resolution or Exacerbation of SARS-CoV-2 in Covid-19: Key Therapeutic Targets?. Front. Immunol..

[8] Zhou R., To K.K.-W., Wong Y.-C., Liu L., Zhou B., Li X., Huang H., Mo Y., Luk T.-Y., Lau T.T.-K. (2020). Acute SARS-CoV-2 Infection Impairs Dendritic Cell and T Cell Responses. Immunity.

[9] Remy K.E., Mazer M., Striker D.A., Ellebedy A.H., Walton A.H., Unsinger J., Blood T.M., Mudd P.A., Yi D.J., Mannion D.A. (2020). Severe immunosuppression and not a cytokine storm characterizes COVID-19 infections. JCI Insight.

[10] Ashraf M.U., Jeong Y., Roh S.-E., Bae Y.-S. (2019). Transendothelial migration (TEM) of in vitro generated dendritic cell vaccine in cancer immunotherapy. Arch. Pharmacal Res..

[11] Kumar S., Jeong Y., Ashraf M.U., Bae Y.-S. (2019). Dendritic Cell-Mediated Th2 Immunity and Immune Disorders. Int. J. Mol. Sci..

[12] Song P., Li W., Xie J., Hou Y., You C. (2020). Cytokine storm induced by SARS-CoV-2. Clin. Chim. Acta.

[13] Zhao J., Yuan Q., Wang H., Liu W., Liao X., Su Y., Wang X., Yuan J., Li T., Li J. (2020). Antibody responses to SARS-CoV-2 in patients of novel coronavirus disease 2019. Clin. Infect. Dis..

[14] Arunachalam P.S., Charles T.P., Joag V., Bollimpelli V.S., Scott M.K.D., Wimmers F., Burton S.L., Labranche C.C., Petitdemange C., Gangadhara S. (2020). T cell-inducing vaccine durably prevents mucosal SHIV infection even with lower neutralizing antibody titers. Nat. Med..

[15] Singh D.D., Han I., Choi E.H., Yadav D.K. (2020). Immunopathology, host-virus genome interactions, and effective vaccine development in SARS-CoV-2. Comput. Struct. Biotechnol. J..

[16] Singh D.D., Han I., Choi E.-H., Yadav D.K. (2020). Recent Advances in Pathophysiology, Drug Development and Future Perspectives of SARS-CoV-2. Front. Cell Dev. Biol..

[17] Saha R.P., Sharma A.R., Singh M.K., Samanta S., Bhakta S., Mandal S., Bhattacharya M., Lee S.-S., Chakraborty C. (2020). Repurposing Drugs, Ongoing Vaccine, and New Therapeutic Development Initiatives Against COVID-19. Front. Pharmacol..

[18] Coronavirus Disease (COVID-19). https://www.google.com/search?q=covid-19+fatality+rate+percentage&oq=Coronavirus-19+fatality+&aqs=chrome.2.69i57j0i10i22i30i457j0i22i30l5.14707j1j15&sourceid=chrome&ie=UTF-8.

[19] Mortality Risk of COVID-19. https://ourworldindata.org/mortality-risk-covid.

[20] O’Leary D.R., Marfin A.A., Montgomery S.P., Kipp A.M., Lehman J.A., Biggerstaff B.J., Elko V.L., Collins P.D., Jones J.E., Campbell G.L. (2004). The Epidemic of West Nile Virus in the United States, 2002. Vector-Borne Zoonotic Dis..

[21] Añez G., Rios M. (2013). Dengue in the United States of America: A Worsening Scenario?. BioMed Res. Int..

[22] Dick O.B., Martín J.L.S., Del Diego J., Montoya R.H., Dayan G.H., Zambrano B. (2012). The History of Dengue Outbreaks in the Americas. Am. J. Trop. Med. Hyg..

[23] Ligon B.L. (2005). Outbreak of Marburg Hemorrhagic Fever in Angola: A Review of the History of the Disease and its Biological Aspects. Semin. Pediatr. Infect. Dis..

[24] Marburg Haemorrhagic Fever in ANGOLA—Update. https://www.who.int/csr/don/2005_03_23/en/.

[25] Renault P., Josseran L., Pierre V. (2008). Chikungunya-related Fatality Rates, Mauritius, India, and Reunion Island. Emerg. Infect. Dis..

[26] Coltart C.E.M., Lindsey B., Ghinai I., Johnson A.M., Heymann D.L. (2017). The Ebola outbreak, 2013–2016: Old lessons for new epidemics. Philos. Trans. R. Soc. B Biol. Sci..

[27] Cardona-Ospina J.A., Henao-SanMartin V., Acevedo-Mendoza W.F., Nasner-Posso K.M., Martínez-Pulgarín D.F., Restrepo-López A., Valencia-Gallego V., Collins M.H., Rodriguez-Morales A.J., Gallego-Valencia V. (2019). Fatal Zika virus infection in the Americas: A systematic review. Int. J. Infect. Dis..

[28] Possas C., Lourenço-De-Oliveira R., Tauil P.L., Pinheiro F.D.P., Pissinatti A., Da Cunha R.V., Freire M., Martins R.M., Homma A. (2018). Yellow fever outbreak in Brazil: The puzzle of rapid viral spread and challenges for immunisation. Memórias Inst. Oswaldo Cruz.

[29] Akpede G.O., Asogun D.A., Okogbenin S.A., Dawodu S.O., Momoh M.O., Dongo A.E., Ike C., Tobin E., Akpede N., Ogbaini-Emovon E. (2019). Caseload and Case Fatality of Lassa Fever in Nigeria, 2001–2018: A Specialist Center’s Experience and Its Implications. Front. Public Health.

[30] Louie J.K., Jean C., Acosta M., Samuel M.C., Mátyás B.T., Schechter R. (2011). A Review of Adult Mortality Due to 2009 Pandemic (H1N1) Influenza A in California. PLoS ONE.

[31] Charu V., Chowell G., Mejia L.S.P., Echevarría-Zuno S., Borja-Aburto V.H., Simonsen L., Miller M.A., Viboud C. (2011). Mortality Burden of the A/H1N1 Pandemic in Mexico: A Comparison of Deaths and Years of Life Lost to Seasonal Influenza. Clin. Infect. Dis..

[32] Fact Check: 2009 Swine Flu Spread Rapidly, But COVID-19 Is More Deadly. https://www.usatoday.com/story/news/factcheck/2020/08/13/fact-check-swine-flu-spread-rapidly-but-not-deadly-covid-19/5577001002/.

[33] Liu D.X., Liang J.Q., Fung T.S. Human Coronavirus-229E, -OC43, -NL63, and -HKU1. https://www.ncbi.nlm.nih.gov/pmc/articles/PMC7204879/.

[34] Sizun J., Yu M., Talbot P. (2000). Survival of human coronaviruses 229E and OC43 in suspension and after drying onsurfaces: A possible source ofhospital-acquired infections. J. Hosp. Infect..

[35] Shin M.D., Shukla S., Chung Y.H., Beiss V., Chan S.K., Ortega-Rivera O.A., Wirth D.M., Chen A., Sack M., Pokorski J.K. (2020). COVID-19 vaccine development and a potential nanomaterial path forward. Nat. Nanotechnol..

[36] Krammer F. (2020). SARS-CoV-2 vaccines in development. Nat. Cell Biol..

[37] Wang H., Zhang Y., Huang B., Deng W., Quan Y., Wang W., Xu W., Zhao Y., Li N., Zhang J. (2020). Development of an Inactivated Vaccine Candidate, BBIBP-CorV, with Potent Protection against SARS-CoV-2. Cell.

[38] Gao Q., Bao L., Mao H., Wang L., Xu K., Yang M., Li Y., Zhu L., Wang N., Lv Z. (2020). Development of an inactivated vaccine candidate for SARS-CoV-2. Science.

[39] Vellozzi C., Burwen D.R., Dobardzic A., Ball R., Walton K., Haber P. (2009). Safety of trivalent inactivated influenza vaccines in adults: Background for pandemic influenza vaccine safety monitoring. Vaccine.

[40] Murdin A.D., Barreto L., Plotkin S. (1996). Inactivated poliovirus vaccine: Past and present experience. Vaccine.

[41] Kusov Y., Elbert L., Nelga I., Grishina G., Dunaevski O., Kharin N., Maslov Y., Drozdov S., Balayan M. (1991). Immunogenicity trial of inactivated hepatitis A virus vaccine in human volunteers. Vaccine.

[42] Furesz J., Scheifele D.W., Palkonyay L. (1995). Safety and effectiveness of the new inactivated hepatitis A virus vaccine. Can. Med. Assoc. J..

[43] Wu W., Liu D., Li K., Nuorti J.P., Nohynek H.M., Xu D., Ye J., Zheng J., Wang H. (2017). Post-marketing safety surveillance for inactivated and live-attenuated Japanese encephalitis vaccines in China, 2008–2013. Vaccine.

[44] Xia S., Duan K., Zhang Y., Zhao D., Zhang H., Xie Z., Li X., Peng C., Zhang Y., Zhang W. (2020). Effect of an Inactivated Vaccine Against SARS-CoV-2 on Safety and Immunogenicity Outcomes: Interim Analysis of 2 Randomized Clinical Trials. JAMA.

[45] Qamar M.T.U., Saleem S., Ashfaq U.A., Bari A., Anwar F., Alqahtani S. (2019). Epitope-based peptide vaccine design and target site depiction against Middle East Respiratory Syndrome Coronavirus: An immune-informatics study. J. Transl. Med..

[46] Watanabe Y., Allen J.D., Wrapp D., McLellan J.S., Crispin M. (2020). Site-specific glycan analysis of the SARS-CoV-2 spike. Science.

[47] Immunogenicity and Safety of a SARS-CoV-2 Inactivated Vaccine in Healthy Adults Aged 18–59 Years: Report of the Randomized, Double-Blind, and Placebo-Controlled Phase 2 Clinical Trial. https://www.medrxiv.org/content/10.1101/2020.07.31.20161216v1.

[48] WHO (2020). DRAFT Landscape of COVID-19 Candidate Vaccines. https://www.who.int/publications/m/item/draft-landscape-of-covid-19-candidate-vaccines.

[49] Minor P.D. (2015). Live attenuated vaccines: Historical successes and current challenges. Virology.

[50] Mohn K.G.-I., Smith I., Sjursen H., Cox R.J. (2018). Immune responses after live attenuated influenza vaccination. Hum. Vaccines Immunother..

[51] Talon J., Salvatore M., O’Neill R.E., Nakaya Y., Zheng H., Muster T., García-Sastre A., Palese P. (2000). Influenza A and B viruses expressing altered NS1 proteins: A vaccine approach. Proc. Natl. Acad. Sci. USA.

[52] Broadbent A.J., Santos C.P., Anafu A., Wimmer E., Mueller S., Subbarao K. (2016). Evaluation of the attenuation, immunogenicity, and efficacy of a live virus vaccine generated by codon-pair bias de-optimization of the 2009 pandemic H1N1 influenza virus, in ferrets. Vaccine.

[53] Griffin D.E. (2018). Measles Vaccine. Viral Immunol..

[54] Plotkin S. (2014). History of vaccination. Proc. Natl. Acad. Sci. USA.

[55] Jimenez-Guardeño J.M., Regla-Nava J.A., Nieto-Torres J.L., DeDiego M.L., Castaño-Rodriguez C., Fernandez-Delgado R., Perlman S., Enjuanes L. (2015). Identification of the Mechanisms Causing Reversion to Virulence in an Attenuated SARS-CoV for the Design of a Genetically Stable Vaccine. PLOS Pathog..

[56] Barrett P.N., Mundt W., Kistner O., Howard M.K. (2009). Vero cell platform in vaccine production: Moving towards cell culture-based viral vaccines. Expert Rev. Vaccines.

[57] (2017). Live attenuated influenza vaccine for children. Drug Ther. Bull..

[58] Armitage E.P., Camara J., Bah S., Forster A.S., Clarke E., Kampmann B., De Silva T.I. (2018). Acceptability of intranasal live attenuated influenza vaccine, influenza knowledge and vaccine intent in The Gambia. Vaccine.

[59] Bahamondez-Canas T.F., Cui Z. (2018). Intranasal immunization with dry powder vaccines. Eur. J. Pharm. Biopharm..

[60] Li R., Lim A., Alonso S. (2011). AttenuatedBordetella pertussisBPZE1 as a live vehicle for heterologous vaccine antigens delivery through the nasal route. Bioeng. Bugs.

[61] Esposito S., Montinaro V., Groppali E., Tenconi R., Semino M., Principi N. (2012). Live attenuated intranasal influenza vaccine. Hum. Vaccines Immunother..

[62] Wang J., Peng Y., Xu H., Cui Z., Williams R. (2020). The COVID-19 Vaccine Race: Challenges and Opportunities in Vaccine Formulation. AAPS PharmSciTech.

[63] Itani R., Tobaiqy M., Al Faraj A. (2020). Optimizing use of theranostic nanoparticles as a life-saving strategy for treating COVID-19 patients. Theranostics.

[64] Xu H., Zhong L., Deng J., Peng J., Dan H., Zeng X., Li T., Chen Q. (2020). High expression of ACE2 receptor of 2019-nCoV on the epithelial cells of oral mucosa. Int. J. Oral Sci..

[65] Sakaguchi W., Kubota N., Shimizu T., Saruta J., Fuchida S., Kawata A., Yamamoto Y., Sugimoto M., Yakeishi M., Tsukinoki K. (2020). Existence of SARS-CoV-2 Entry Molecules in the Oral Cavity. Int. J. Mol. Sci..

[66] Gurwith M., Condit R.C., Excler J.-L., Robertson J.S., Kim D., Fast P.E., Drew S., Wood D., Klug B., Whelan M. (2020). Brighton Collaboration Viral Vector Vaccines Safety Working Group (V3SWG) standardized template for collection of key information for benefit-risk assessment of live-attenuated viral vaccines. Vaccine.

[67] Halsey N.A., Talaat K.R., Greenbaum A., Mensah E., Dudley M.Z., Proveaux T., Salmon D.A. (2015). The safety of influenza vaccines in children: An Institute for Vaccine Safety white paper. Vaccine.

[68] Zheng Z., Diaz-Arévalo D., Guan H., Zeng M. (2018). Noninvasive vaccination against infectious diseases. Hum. Vaccines Immunother..

[69] Bhandari R., Khanna G., Kuhad A. (2021). Pharmacological insight into potential therapeutic agents for the deadly Covid-19 pandemic. Eur. J. Pharmacol..

[70] Seo S.H., Jang Y. (2020). Cold-Adapted Live Attenuated SARS-Cov-2 Vaccine Completely Protects Human ACE2 Transgenic Mice from SARS-Cov-2 Infection. Vaccines.

[71] Sims A.C., Baric R.S., Yount B., Burkett S.E., Collins P.L., Pickles R.J. (2005). Severe Acute Respiratory Syndrome Coronavirus Infection of Human Ciliated Airway Epithelia: Role of Ciliated Cells in Viral Spread in the Conducting Airways of the Lungs. J. Virol..

[72] Nascimento I., Leite L. (2012). Recombinant vaccines and the development of new vaccine strategies. Braz. J. Med. Biol. Res..

[73] Mardanova E.S., Ravin N.V. (2018). Plant-produced Recombinant Influenza a Vaccines Based on the M2e Peptide. Curr. Pharm. Des..

[74] Barnard R.T. (2010). Recombinant vaccines. Expert Rev. Vaccines.

[75] Du L., Zhang X., Liu J., Jiang S. (2011). Protocol for Recombinant RBD-based SARS Vaccines: Protein Preparation, Animal Vaccination and Neutralization Detection. J. Vis. Exp..

[76] Huber V.C. (2013). Influenza vaccines: From whole virus preparations to recombinant protein technology. Expert Rev. Vaccines.

[77] Humphreys I.R., Sebastian S. (2017). Novel viral vectors in infectious diseases. Immunology.

[78] Draper S.J., Heeney J.L. (2009). Viruses as vaccine vectors for infectious diseases and cancer. Nat. Rev. Genet..

[79] Cox M.M., Hollister J.R. (2009). FluBlok, a next generation influenza vaccine manufactured in insect cells. Biologicals.

[80] Henry C., Palm A.-K.E., Utset H.A., Huang M., Ho I.Y., Zheng N.-Y., Fitzgerald T., Neu K.E., Chen Y.-Q., Krammer F. (2019). Monoclonal Antibody Responses after Recombinant Hemagglutinin Vaccine versus Subunit Inactivated Influenza Virus Vaccine: A Comparative Study. J. Virol..

[81] Cox M.M., Patriarca P.A., Treanor J. (2008). FluBlok, a recombinant hemagglutinin influenza vaccine. Influ. Other Respir. Viruses.

[82] Xie J., He Y., Shen B. (2017). Ontology-Based Vaccine Adverse Event Representation and Analysis. Adv. Exp. Med. Biol..

[83] Cox M.M.J., Izikson R., Post P., Dunkle L.M. (2015). Safety, efficacy, and immunogenicity of Flublok in the prevention of seasonal influenza in adults. Ther. Adv. Vaccines.

[84] Amanat F., Stadlbauer D., Strohmeier S., Nguyen T.H.O., Chromikova V., McMahon M., Jiang K., Arunkumar G.A., Jurczyszak D., Polanco J. (2020). A serological assay to detect SARS-CoV-2 seroconversion in humans. Nat. Med..

[85] Smith C.L., Mirza F., Pasquetto V., Tscharke D.C., Palmowski M.J., Dunbar P.R., Sette A., Harris A.L., Cerundolo V. (2005). Immunodominance of Poxviral-Specific CTL in a Human Trial of Recombinant-Modified Vaccinia Ankara. J. Immunol..

[86] Thomson S.A., Elliott S.L., Sherritt M.A., Sproat K.W., Coupar B.E., Scalzo A.A., Forbes C.A., Ladhams A.M., Mo X.Y., Tripp R.A. (1996). Recombinant polyepitope vaccines for the delivery of multiple CD8 cytotoxic T cell epitopes. J. Immunol..

[87] Smith C.L., Dunbar P.R., Mirza F., Palmowski M.J., Shepherd D., Gilbert S.C., Coulie P., Schneider J., Hoffman E., Hawkins R. (2004). Recombinant modified vaccinia Ankara primes functionally activated CTL specific for a melanoma tumor antigen epitope in melanoma patients with a high risk of disease recurrence. Int. J. Cancer.

[88] Liu Z., Xu W., Xia S., Gu C., Wang X., Wang Q., Zhou J., Wu Y., Cai X., Qu D. (2020). RBD-Fc-based COVID-19 vaccine candidate induces highly potent SARS-CoV-2 neutralizing antibody response. Signal Transduct. Target. Ther..

[89] Tai W., He L., Zhang X., Pu J., Voronin D., Jiang S., Zhou Y., Du L. (2020). Characterization of the receptor-binding domain (RBD) of 2019 novel coronavirus: Implication for development of RBD protein as a viral attachment inhibitor and vaccine. Cell. Mol. Immunol..

[90] Dai L., Zheng T., Xu K., Han Y., Xu L., Huang E., An Y., Cheng Y., Li S., Liu M. (2020). A Universal Design of Betacoronavirus Vaccines against COVID-19, MERS, and SARS. Cell.

[91] Ravichandran S., Coyle E.M., Klenow L., Tang J., Grubbs G., Liu S., Wang T., Golding H., Khurana S. (2020). Antibody signature induced by SARS-CoV-2 spike protein immunogens in rabbits. Sci. Transl. Med..

[92] Dutta A.K. (2020). Vaccine Against Covid-19 Disease—Present Status of Development. Indian J. Pediatr..

[93] Vogel F.R., Sarver N. (1995). Nucleic acid vaccines. Clin. Microbiol. Rev..

[94] Pardi N., Hogan M.J., Porter F.W., Weissman D. (2018). mRNA vaccines—A new era in vaccinology. Nat. Rev. Drug Discov..

[95] Jackson N.A.C., Kester K.E., Casimiro D., Gurunathan S., DeRosa F. (2020). The promise of mRNA vaccines: A biotech and industrial perspective. NPJ Vaccines.

[96] Vogel A.B., Lambert L., Kinnear E., Busse D., Erbar S., Reuter K.C., Wicke L., Perkovic M., Beissert T., Haas H. (2018). Self-Amplifying RNA Vaccines Give Equivalent Protection against Influenza to mRNA Vaccines but at Much Lower Doses. Mol. Ther..

[97] Corbett K.S., Edwards D.K., Leist S.R., Abiona O.M., Boyoglu-Barnum S., Gillespie R.A., Himansu S., Schäfer A., Ziwawo C.T., DiPiazza A.T. (2020). SARS-CoV-2 mRNA vaccine design enabled by prototype pathogen preparedness. Nat. Cell Biol..

[98] Laczkó D., Hogan M.J., Toulmin S.A., Hicks P., Lederer K., Gaudette B.T., Castaño D., Amanat F., Muramatsu H., Oguin T.H. (2020). A Single Immunization with Nucleoside-Modified mRNA Vaccines Elicits Strong Cellular and Humoral Immune Responses against SARS-CoV-2 in Mice. Immunity.

[99] Corbett K.S., Flynn B., Foulds K.E., Francica J.R., Boyoglu-Barnum S., Werner A.P., Flach B., O’Connell S., Bock K.W., Minai M. (2020). Evaluation of the mRNA-1273 Vaccine against SARS-CoV-2 in Nonhuman Primates. N. Engl. J. Med..

[100] Lu J., Lu G., Tan S., Xia J., Xiong H., Yu X., Qi Q., Yu X., Li L., Yu H. (2020). A COVID-19 mRNA vaccine encoding SARS-CoV-2 virus-like particles induces a strong antiviral-like immune response in mice. Cell Res..

[101] Petsch B., Schnee M., Vogel A.B., Lange E., Hoffmann B., Voss D., Schlake T., Thess A., Kallen K.-J., Stitz L. (2012). Protective efficacy of in vitro synthesized, specific mRNA vaccines against influenza A virus infection. Nat. Biotechnol..

[102] Chahal J.S., Khan O.F., Cooper C.L., McPartlan J.S., Tsosie J.K., Tilley L.D., Sidik S.M., Lourido S., Langer R., Bavari S. (2016). Dendrimer-RNA nanoparticles generate protective immunity against lethal Ebola, H1N1 influenza, and Toxoplasma gondii challenges with a single dose. Proc. Natl. Acad. Sci. USA.

[103] Schnee M., Vogel A.B., Voss D., Petsch B., Baumhof P., Kramps T., Stitz L. (2016). An mRNA Vaccine Encoding Rabies Virus Glycoprotein Induces Protection against Lethal Infection in Mice and Correlates of Protection in Adult and Newborn Pigs. PLoS Negl. Trop. Dis..

[104] Bahl K., Senn J.J., Yuzhakov O., Bulychev A., Brito L.A., Hassett K.J., Laska M.E., Smith M., Almarsson Ö., Thompson J. (2017). Preclinical and Clinical Demonstration of Immunogenicity by mRNA Vaccines against H10N8 and H7N9 Influenza Viruses. Mol. Ther..

[105] Pardi N., Hogan M.J., Pelc R.S., Muramatsu H., Andersen H., DeMaso C.R., Dowd K.A., Sutherland L.L., Scearce R.M., Parks R. (2017). Zika virus protection by a single low-dose nucleoside-modified mRNA vaccination. Nature.

[106] FDA Takes Additional Action in Fight against COVID-19 by Issuing Emergency Use Authorization for Second COVID-19 Vaccine. https://www.fda.gov/news-events/press-announcements/fda-takes-additional-action-fight-against-covid-19-issuing-emergency-use-authorization-second-covid.

[107] Mahase E. (2020). Covid-19: Pfizer and BioNTech submit vaccine for US authorisation. BMJ.

[108] Jackson L.A., Anderson E.J., Rouphael N.G., Roberts P.C., Makhene M., Coler R.N., McCullough M.P., Chappell J.D., Denison M.R., Stevens L.J. (2020). An mRNA Vaccine against SARS-CoV-2—Preliminary Report. N. Engl. J. Med..

[109] Mulligan M.J., Lyke K.E., Kitchin N., Absalon J., Gurtman A., Lockhart S., Neuzil K., Raabe V., Bailey R., Swanson K.A. (2020). Phase I/II study of COVID-19 RNA vaccine BNT162b1 in adults. Nature.

[110] Hobernik D., Bros M. (2018). DNA Vaccines—How Far From Clinical Use?. Int. J. Mol. Sci..

[111] Explained: Why RNA Vaccines for Covid-19 Raced to the Front of the Pack. https://news.mit.edu/2020/rna-vaccines-explained-covid-19-1211.

[112] Zeng C., Hou X., Yan J., Zhang C., Li W., Zhao W., Du S., Dong Y. (2020). Leveraging mRNAs Sequences to Express SARS-CoV-2 Antigens in vivo. bioRxiv.

[113] Mutant Coronavirus in the United Kingdom Sets Off Alarms, But Its Importance Remains Unclear. https://www.sciencemag.org/news/2020/12/mutant-coronavirus-united-kingdom-sets-alarms-its-importance-remains-unclear.

[114] Arcturus Therapeutics and Duke-NUS Medical School Partner to Develop a Coronavirus (COVID-19) Vaccine using STARR™ Technology. https://ir.arcturusrx.com/news-releases/news-release-details/arcturus-therapeutics-and-duke-nus-medical-school-partner.

[115] Walsh E.E., Frenck R.W., Falsey A.R., Kitchin N., Absalon J., Gurtman A., Lockhart S., Neuzil K., Mulligan M.J., Bailey R. (2020). Safety and Immunogenicity of Two RNA-Based Covid-19 Vaccine Candidates. N. Engl. J. Med..

[116] Zeng C., Hou X., Yan J., Zhang C., Li W., Zhao W., Du S., Dong Y. (2020). Leveraging mRNA Sequences and Nanoparticles to Deliver SARS-CoV-2 Antigens In Vivo. Adv. Mater..

[117] CanSino’s Coronavirus Vaccine Candidate Approved for Military Use in China. https://www.cnbc.com/2020/06/29/cansinos-coronavirus-vaccine-candidate-approved-for-military-use-in-china.html.

[118] Zhu F.-C., Guan X.-H., Li Y.-H., Huang J.-Y., Jiang T., Hou L.-H., Li J.-X., Yang B.-F., Wang L., Wang W.-J. (2020). Immunogenicity and safety of a recombinant adenovirus type-5-vectored COVID-19 vaccine in healthy adults aged 18 years or older: A randomised, double-blind, placebo-controlled, phase 2 trial. Lancet.

[119] Zhu F.-C., Li Y.-H., Guan X.-H., Hou L.-H., Wang W.-J., Li J.-X., Wu S.-P., Wang B.-S., Wang Z., Wang L. (2020). Safety, tolerability, and immunogenicity of a recombinant adenovirus type-5 vectored COVID-19 vaccine: A dose-escalation, open-label, non-randomised, first-in-human trial. Lancet.

[120] Van Doremalen N., Lambe T., Spencer A., Belij-Rammerstorfer S., Purushotham J.N., Port J.R., Avanzato V.A., Bushmaker T., Flaxman A., Ulaszewska M. (2020). ChAdOx1 nCoV-19 vaccine prevents SARS-CoV-2 pneumonia in rhesus macaques. Nature.

[121] Folegatti P.M., Ewer K.J., Aley P.K., Angus B., Becker S., Belij-Rammerstorfer S., Bellamy D., Bibi S., Bittaye M., Clutterbuck E.A. (2020). Safety and immunogenicity of the ChAdOx1 nCoV-19 vaccine against SARS-CoV-2: A preliminary report of a phase 1/2, single-blind, randomised controlled trial. Lancet.

[122] Logunov D.Y., Dolzhikova I.V., Zubkova O.V., Tukhvatullin A.I., Shcheblyakov D.V., Dzharullaeva A.S., Grousova D.M., Erokhova A.S., Kovyrshina A.V., Botikov A.G. (2020). Safety and immunogenicity of an rAd26 and rAd5 vector-based heterologous prime-boost COVID-19 vaccine in two formulations: Two open, non-randomised phase 1/2 studies from Russia. Lancet.

[123] Mercado N.B., Zahn R., Wegmann F., Loos C., Chandrashekar A., Yu J., Liu J., Peter L., Mcmahan K., Tostanoski L.H. (2020). Single-shot Ad26 vaccine protects against SARS-CoV-2 in rhesus macaques. Nature.

[124] Smith T.R.F., Patel A., Ramos S., Elwood D., Zhu X., Yan J., Gary E.N., Walker S.N., Schultheis K., Purwar M. (2020). Immunogenicity of a DNA vaccine candidate for COVID-19. Nat. Commun..

[125] Zhang N.-N., Li X.-F., Deng Y.-Q., Zhao H., Huang Y.-J., Yang G., Huang W.-J., Gao P., Zhou C., Zhang R.-R. (2020). A Thermostable mRNA Vaccine against COVID-19. Cell.

[126] Martin C., Lowery D. (2020). mRNA vaccines: Intellectual property landscape. Nat. Rev. Drug Discov..

[127] McKay P.F., Hu K., Blakney A.K., Samnuan K., Brown J.C., Penn R., Zhou J., Bouton C.R., Rogers P., Polra K. (2020). Self-amplifying RNA SARS-CoV-2 lipid nanoparticle vaccine candidate induces high neutralizing antibody titers in mice. Nat. Commun..

[128] Rabaan A.A., Al-Ahmed S.H., Sah R., Al-Tawfiq J.A., Al-Qaaneh A.M., Al-Jamea L.H., Woodman A., Al-Qahtani M., Haque S., Harapan H. (2020). Recent advances in vaccine and immunotherapy for COVID-19. Hum. Vaccines Immunother..

[129] Sayedahmed E.E., Elkashif A., Alhashimi M., Sambhara S., Mittal S.K. (2020). Adenoviral Vector-Based Vaccine Platforms for Developing the Next Generation of Influenza Vaccines. Vaccines.

[130] Sharpe H.R., Gilbride C., Allen E., Belij-Rammerstorfer S., Bissett C., Ewer K., Lambe T. (2020). The early landscape of coronavirus disease 2019 vaccine development in the UK and rest of the world. Immunology.

[131] Poland A.G., Ovsyannikova I.G., Kennedy R.B. (2020). SARS-CoV-2 immunity: Review and applications to phase 3 vaccine candidates. Lancet.

[132] AstraZeneca’s COVID-19 Vaccine Authorised in Five Other Countries. https://www.astrazeneca.com/content/astraz/media-centre/press-releases/2021/serum-institute-of-india-obtains-emergency-use-authorisation-in-india-for-astrazenecas-covid-19-vaccine.html.

[133] Xia S., Zhang Y., Wang Y., Wang H., Yang Y., Gao G.F., Tan W., Wu G., Xu M., Lou Z. (2020). Safety and immunogenicity of an inactivated SARS-CoV-2 vaccine, BBIBP-CorV: A randomised, double-blind, placebo-controlled, phase 1/2 trial. Lancet Infect. Dis..

[134] China Injects Hundreds of Thousands With Experimental Covid-19 Vaccines. https://www.wsj.com/articles/china-injects-hundreds-of-thousands-with-experimental-covid-19-vaccines-11599834029.

[135] Russia’s Claim of a Successful COVID-19 Vaccine Doesn’t Pass the ‘Smell Test,’ Critics Say. https://www.sciencemag.org/news/2020/11/russia-s-claim-successful-covid-19-vaccine-doesn-t-pass-smell-test-critics-say.

[136] Keech C., Albert G., Cho I., Robertson A., Reed P., Neal S., Plested J.S., Zhu M., Cloney-Clark S., Zhou H. (2020). Phase 1–2 Trial of a SARS-CoV-2 Recombinant Spike Protein Nanoparticle Vaccine. N. Engl. J. Med..

[137] Guebre-Xabier M., Patel N., Tian J.-H., Zhou B., Maciejewski S., Lam K., Portnoff A.D., Massare M.J., Frieman M.B., Piedra P.A. (2020). NVX-CoV2373 vaccine protects cynomolgus macaque upper and lower airways against SARS-CoV-2 challenge. Vaccine.

[138] Tian J.-H., Patel N., Haupt R., Zhou H., Weston S., Hammond H., Logue J., Portnoff A.D., Norton J., Guebre-Xabier M. (2021). SARS-CoV-2 spike glycoprotein vaccine candidate NVX-CoV2373 immunogenicity in baboons and protection in mice. Nat. Commun..

[139] Sinovac’s Covid-19 Vaccine Gets Emergency Use Approval in China. https://www.pharmaceutical-technology.com/news/sinovac-vaccine-emergency-use.

[140] Johnson & Johnson Prepares to Resume Phase 3 ENSEMBLE Trial of Its Janssen COVID-19 Vaccine Candidate in the U.S. https://www.jnj.com/our-company/johnson-johnson-prepares-to-resume-phase-3-ensemble-trial-of-its-janssen-covid-19-vaccine-candidate-in-the-us.

[141] Sadoff J., Le Gars M., Shukarev G., Heerwegh D., Truyers C., De Groot A.M., Stoop J., Tete S., Van Damme W., Leroux-Roels I. (2021). Interim Results of a Phase 1–2a Trial of Ad26.COV2.S Covid-19 Vaccine. N. Engl. J. Med..

[142] Walsh E.E., Frenck R., Falsey A.R., Kitchin N., Absalon J., Gurtman A., Lockhart S., Neuzil K., Mulligan M.J., Bailey R. (2020). RNA-Based COVID-19 Vaccine BNT162b2 Selected for a Pivotal Efficacy Study. medRxiv.

[143] Oliver S.E., Gargano J.W., Marin M., Wallace M., Curran K.G., Chamberland M., McClung N., Campos-Outcalt D., Morgan R.L., Mbaeyi S. (2020). The Advisory Committee on Immunization Practices’ Interim Recommendation for Use of Pfizer-BioNTech COVID-19 Vaccine—United States, December 2020. MMWR Morb. Mortal. Wkly. Rep..

[144] U.K. Approves Pfizer’s Covid-19 Vaccine, Putting Pressure on FDA. https://www.statnews.com/2020/12/02/u-k-approves-pfizers-covid-19-vaccine-putting-pressure-on-fda/#:~:text=U.K.%20approves%20Pfizer’s%20Covid%2D19%20vaccine%2C%20putting%20pressure%20on%20FDA,-By%20Matthew%20Herper&text=The%20United%20Kingdom%20on,swiftly%20to%20do%20the%20same.

[145] BioNTech, Pfizer, and Fosun Pharma—BNT162b2. https://www.genengnews.com/covid-19-candidates/biontech-pfizer-and-fosun-pharma-bnt162/.

[146] Mahase E. (2020). Covid-19: Pfizer vaccine efficacy was 52% after first dose and 95% after second dose, paper shows. BMJ.

[147] Polack F.P., Thomas S.J., Kitchin N., Absalon J., Gurtman A., Lockhart S., Perez J.L., Marc G.P., Moreira E.D., Zerbini C. (2020). Safety and Efficacy of the BNT162b2 mRNA Covid-19 Vaccine. N. Engl. J. Med..

[148] Mahase E. (2020). Covid-19: People with history of significant allergic reactions should not receive Pfizer vaccine, says regulator. BMJ.

[149] Anderson E.J., Rouphael N.G., Widge A.T., Jackson L.A., Roberts P.C., Makhene M., Chappell J.D., Denison M.R., Stevens L.J., Pruijssers A.J. (2020). Safety and Immunogenicity of SARS-CoV-2 mRNA-1273 Vaccine in Older Adults. N. Engl. J. Med..

[150] mRNA-1273 Clinical Development Program. https://investors.modernatx.com/static-files/34f97bb2-d89a-45e4-a770-cae0591fa807.

[151] Nichol A.A. (2020). Potential Implications of Testing an Experimental mRNA-Based Vaccine during an Emerging Infectious Disease Pandemic. Am. J. Bioeth..

[152] Moderna Announces Primary Efficacy Analysis in Phase 3 COVE Study for Its COVID-19 Vaccine Candidate and Filing Today with U.S FDA for Emergency Use Authorization. https://investors.modernatx.com/news-releases/news-release-details/moderna-announces-primary-efficacy-analysis-phase-3-cove-study.

[153] Hellfritzsch M., Scherließ R. (2019). Mucosal Vaccination via the Respiratory Tract. Pharmaceutics.

[154] Brandtzaeg P. (2011). Potential of Nasopharynx-associated Lymphoid Tissue for Vaccine Responses in the Airways. Am. J. Respir. Crit. Care Med..

[155] Digestive System Is a Potential Route of COVID-19: An Analysis of Single-Cell Coexpression Pattern of Key Proteins in Viral Entry Process. https://gut.bmj.com/content/69/6/1010.

[156] Hou Y.J., Okuda K., Edwards C.E., Martinez D.R., Asakura T., Dinnon K.H., Kato T., Lee R.E., Yount B.L., Mascenik T.M. (2020). SARS-CoV-2 Reverse Genetics Reveals a Variable Infection Gradient in the Respiratory Tract. Cell.

[157] Ramirez J.E.V., Sharpe L.A., Peppas N.A. (2017). Current state and challenges in developing oral vaccines. Adv. Drug Deliv. Rev..

[158] Choudhry A., Mathena J., Albano J.D., Yacovone M., Collins L. (2016). Safety evaluation of adenovirus type 4 and type 7 vaccine live, oral in military recruits. Vaccine.

[159] Vaxart’s Oral COVID-19 Tablet Vaccine to Enter Clinical Trials. https://www.biopharma-reporter.com/Article/2020/09/15/Vaxart-First-tablet-COVID-19-vaccine-to-enter-clinical-trials.

[160] Pre-Clinical Studies of a Recombinant Adenoviral Mucosal Vaccine to Prevent SARS-CoV-2 Infection. https://www.biorxiv.org/content/10.1101/2020.09.04.283853v1.

[161] Chumakov K., Benn C.S., Aaby P., Kottilil S., Gallo R. (2020). Can existing live vaccines prevent COVID-19?. Science.

[162] Vaxart Has a Development Program Focused on Prophylactic and Therapeutic Vaccines in Multiple Indications. https://vaxart.com/pipeline/.

[163] Stabilitech Biopharma Announces Name Change to iosBio. https://www.globenewswire.com/news-release/2020/09/30/2101099/0/en/Stabilitech-Biopharma-announces-name-change-to-iosBio.html.

[164] ORAPRO-COVID-19 Vaccine Capsules—Thermally Stable and Orally Administered. https://www.stabilitech.com/orapro-covid-19.

[165] Grassly N.C., Jafari H., Bahl S., Sethi R., Deshpande J.M., Wolff C., Sutter R.W., Aylward R.B. (2012). Waning Intestinal Immunity After Vaccination With Oral Poliovirus Vaccines in India. J. Infect. Dis..

[166] Han S.-S., Lee J., Jung Y., Kang M.-H., Hong J.-H., Cha M.-S., Park Y.-J., Lee E., Yoon C.-H., Bae Y.-S. (2015). Development of oral CTL vaccine using a CTP-integrated Sabin 1 poliovirus-based vector system. Vaccine.

[167] Bandyopadhyay A.S., Garon J., Seib K., Orenstein W.A. (2015). Polio vaccination: Past, present and future. Future Microbiol..

[168] Nomoto A., Omata T., Toyoda H., Kuge S., Horie H., Kataoka Y., Genba Y., Nakano Y., Imura N. (1982). Complete nucleotide sequence of the attenuated poliovirus Sabin 1 strain genome. Proc. Natl. Acad. Sci. USA.

[169] Xiao Y., Daniell H. (2017). Long-term evaluation of mucosal and systemic immunity and protection conferred by different polio booster vaccines. Vaccine.

[170] Okayasu H., Sutter R.W., Czerkinsky C., Ogra P.L. (2011). Mucosal immunity and poliovirus vaccines: Impact on wild poliovirus infection and transmission. Vaccine.

